# Hot-band absorption of indocyanine green for advanced anti-stokes fluorescence bioimaging

**DOI:** 10.1038/s41377-021-00627-1

**Published:** 2021-09-13

**Authors:** Jing Zhou, Xiaoxiao Fan, Di Wu, Jie Liu, Yuhuang Zhang, Zikang Ye, Dingwei Xue, Mubin He, Liang Zhu, Zhe Feng, Andrey N. Kuzmin, Wen Liu, Paras N. Prasad, Jun Qian

**Affiliations:** 1 State Key Laboratory of Modern Optical Instrumentations, Centre for Optical and Electromagnetic Research, College of Optical Science and Engineering, International Research Center for Advanced Photonics, Zhejiang University, Hangzhou, 310058 China; 2 Sir Run-Run Shaw Hospital, School of Medicine, Zhejiang University, Hangzhou, 310016 China; 3 Key Laboratory of Flexible Electronics (KLOFE) Institute of Advanced Materials (IAM), Nanjing Tech University (Nanjing Tech), Nanjing, 211800 China; 4 Department of Chemistry, Zhejiang University, Hangzhou, 310058 China; 5 Interdisciplinary Institute of Neuroscience and Technology (ZIINT), College of Biomedical Engineering and Instrument Science, Zhejiang University, Hangzhou, 310027 China; 6 Institute for Lasers, Photonics, and Biophotonics, Department of Chemistry, University at Buffalo, State University of New York, Buffalo, NY 14260 USA; 7 Key Laboratory of Optical Information Detection and Display Technology of Zhejiang, Zhejiang Normal University, Jinhua, 321004 China

**Keywords:** Imaging and sensing, Biophotonics

## Abstract

Bright anti-Stokes fluorescence (ASF) in the first near-infrared spectral region (NIR-I, 800 nm–900 nm) under the excitation of a 915 nm continuous wave (CW) laser, is observed in Indocyanine Green (ICG), a dye approved by the Food and Drug Administration for clinical use. The dependence of fluorescence intensity on excitation light power and temperature, together with fluorescence lifetime measurement, establish this ASF to be originated from absorption from a thermally excited vibrational level (hot-band absorption), as shown in our experiments, which is stronger than the upconversion fluorescence from widely-used rare-earth ion doped nanoparticles. To test the utility of this ASF NIR-I probe for advanced bioimaging, we successively apply it for biothermal sensing, cerebral blood vessel tomography and blood stream velocimetry. Moreover, in combination with L1057 nanoparticles, which absorb the ASF of ICG and emit beyond 1100 nm, these two probes generate multi-mode images in two fluorescent channels under the excitation of a single 915 nm CW laser. One channel is used to monitor two overlapping organs, urinary system & blood vessel of a live mouse, while the other shows urinary system only. Using in intraoperative real-time monitoring, such multi-mode imaging method can be beneficial for visual guiding in anatomy of the urinary system to avoid any accidental injury to the surrounding blood vessels during surgery.

## Abstract (web summary)

Hot-band absorption based anti-Stokes fluorescence of ICG for evaluating the thermal state of the tumor and multi-organ imaging.

## Graphical abstract

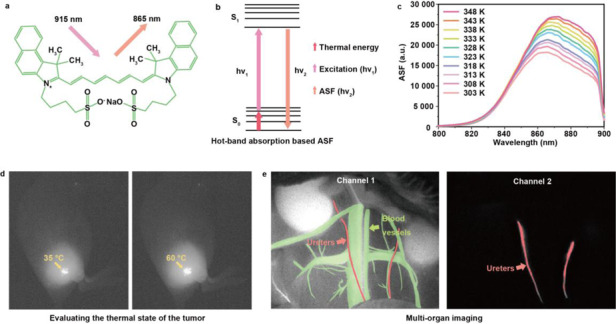

## Introduction

Anti-Stokes luminescence is an optical process, wherein the absorption of long-wavelength photons produces short-wavelength emission light. The anti-Stokes fluorescence (ASF) excitation provides unparallel benefits for biomedical applications. Owing to the longer wavelength, and therefore, lower scattering in tissues, the excitation light of ASF can penetrate deeper into biological samples. Longer wavelength excitation light also has lower photon energy, generating weaker autofluorescence of biological tissues, which is able to avoid the background interference during bioimaging to a certain extent.

There are four processes that can produce ASF - (i) direct multiphoton absorption (MPA) process, (ii) upconversion process based on multistep absorption through intermediate energy levels, (iii) thermally activated delayed fluorescence (TADF) process, and (iv) hot-band absorption (HBA) process^1,2^. The MPA process occurring in materials such as dye molecules, aggregation-induced emission nanoparticles, quantum dots, etc., have been routinely used for multiphoton microscopy^3–5^. The occurrence of MPA emission generally requires extremely high excitation intensity and is usually achieved by using expensive femto- or pico-second pulsed lasers. Upconversion processes in rare-earth ion doped materials, or triplet-triplet annihilation-based upconversion^6^, can be obtained by using inexpensive continuous wave (CW) diode lasers. However, the absorption cross-section of rare-earth ion doped materials is relatively small, resulting in low upconversion efficiency^7^. Triplet-triplet annihilation-based metal complex-organic compound systems have stronger absorption and higher quantum efficiency to be more efficient upconverters than rare-earth ion doped materials^8,9^. Unfortunately, the photostability of triplet-triplet annihilation-based upconverters is relatively low due to strong quenching processes caused by molecular oxygen^10^. TADF and HBA processes in organic molecules also excited by the CW laser are attractive anti-Stokes processes. What’s more, the potential of them to provide information about temperature in excited volume, makes them more attractive for application in bioimaging.

In our study, an organic small-molecule dye, Indocyanine Green (ICG), approved by the Food and Drug Administration (FDA) for clinical use^11,12^, was found to produce bright ASF in the first near-infrared (NIR-I, 800–900 nm) spectral region under CW laser excitation at 915 nm. Excitation power dependence, temperature dependence, and lifetime measurement were carried out to establish that this ASF is originated from HBA, and thus biothermal sensing imaging was achieved. We show that this ASF is much stronger than typical upconversion fluorescence in rare-earth ion doped nanoparticles (UCNPs) excited at 980 nm, with negligible thermal damage to biological tissues. Deep volume tomography of cerebral blood vessels and measurement of the blood flow velocity of mice were performed by using the NIR-I ASF of ICG. By combining ICG with fluorescent organic polymer dots L1057, we proposed a concept of real-time in vivo multi-mode imaging enabling high contrast and selective detection of adjacent tissue elements. This approach was validated in urinary organs and blood vessels under a single light source excitation, 915 nm CW laser. This new imaging technique can be helpful for intraoperative real-time monitoring and avoiding accidental surgery injury.

## Results

### ICG’s ASF characterization

**Fig. 1:**
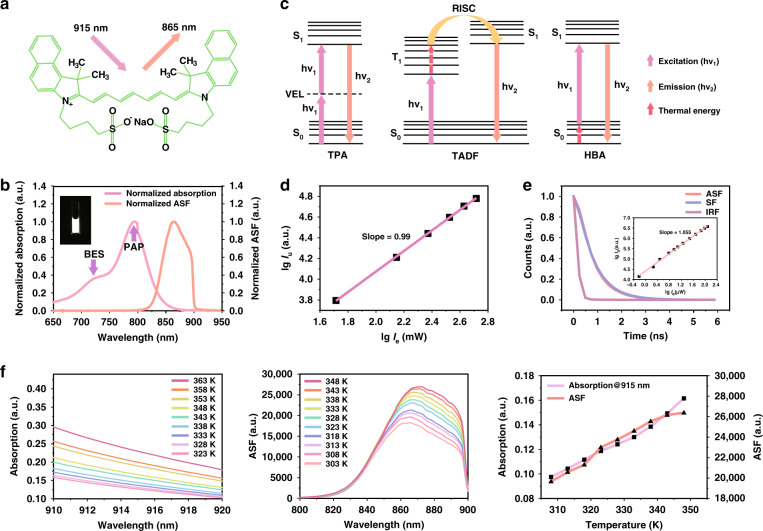
ASF characterization of ICG in DMSO. **a** The molecular structure of ICG. **b** The normalized absorption and ASF ( <900 nm) spectra of ICG. Insert: the ASF image of ICG (0.1 mg mL^−1^), excitation: 915 nm CW laser (26.8 mW cm^−2^), fluorescence collection range: 800–900 nm, exposure time: 25 ms. **c** Schematic illustration of different ASF processes in organic molecules. **d** A logarithmic plot showing the power dependence of ICG’s ASF at 865 nm on the excitation light, 915 nm CW laser. **e** Lifetimes of ASF and SF of ICG (2 mg mL^−1^). Excitation: 915 nm fs pulsed laser for ASF, 750 nm fs pulsed laser for SF; fluorescence collection range: 850–900 nm. Insert: the power dependence of ICG’s ASF intensity on the 915 nm fs pulsed laser intensity. **f** Temperature dependence of ICG’s absorption spectra (910–920 nm) and ASF spectra (800–900 nm) excited by the 915 nm CW laser. The temperature dependence of ICG’s absorption at 915 nm, as well as the ASF intensity excited by the CW laser at this wavelength, is also plotted. Abbreviations: PAP principal absorption peak, BES blue edge shoulder, TPA two-photon absorption, VEL virtual energy level, RISC reverse intersystem crossing, IRF instrument response function

ICG is a small-molecule dye (Fig. 1a). From the absorption spectrum of ICG in dimethyl sulfoxide (DMSO) (Fig. 1b), it can be seen that its principal absorption peak is at 794 nm, which corresponds to the transition from the lowest vibrational energy level (v_0_) of the ground state (S_0_) to the lowest vibrational energy level (v_0_’) of the excited state (S_1_). The blue edge shoulder at 720 nm is associated with the v_0_ → v_1_’ of the S_0_ → S_1_ electronic transition, where v_1_’ is a higher vibrational energy level of S_1_^13^. The ASF spectrum of ICG was measured under the CW laser excitation at 915 nm, which is at the long-wavelength wing of the absorption spectrum. As previously reported, the full-spectrum quantum efficiency of ICG dissolved in DMSO is 13%^14^, giving an estimation for the ASF quantum efficiency between 800–900 nm as ~8%.

To establish the mechanism of ASF in ICG, we measured the excitation power dependence of the ASF intensity, and the ASF lifetime. The fluorescence spectra of 0.1 mg mL^−1^ ICG in DMSO were measured at different excitation powers, and the result of measurement is shown as a logarithmic power dependence (Fig. 1d), where the slope coefficient (0.99) of the fitting line is close to 1, indicating the linear mechanism of ASF.

The ASF and Stokes fluorescence (SF) decay curves of ICG in DMSO were measured using a time-correlated single-photon counting (TCSPC) technique under excitation by femtosecond (fs) pulsed lasers (Fig. 1e). The fluorescence lifetimes of both excitation channels are almost the same, ~0.83 ns. It is worth noting that when the average power of 915 nm fs pulsed laser was less than 120 μW after passing through the objective, only a linear optical process (slope = 1.055) was involved to generate ASF (insert in Fig. 1e). For measuring the ASF lifetime, the average power of 915 nm fs pulsed laser was 84 μW. Thus, only a linear process was involved in the measurement of ASF lifetime, which is similar to that under 915 nm CW laser excitation. TADF lifetime has an order of microseconds due to long-lived triplet states^15–18^, we conclude that the ASF mechanism in ICG should not be TADF.

In general, the HBA fluorescence and TADF mechanisms are similar, both of which involve thermal activation (Fig. 1c)^19–25^. However, in the HBA process, the electron of a dye molecule absorbs the photon from the upper, thermally populated, vibrational level of the ground state. After decaying from the excited state, the electron again populates the ground state, but in the lower vibrational level, thus emitting the photon with higher energy compared to that of absorbed initially. The thermal equilibrium approximation vibration population, governing the ASF process, satisfies the Boltzmann distribution^26^:

1 $$\frac{{n_i}}{{n_0}} = e^{ - E_i/k_BT}$$

where *n*_*0*_ is the molecular population of the lowest vibrational energy level in the ground state, while *n*_*i*_ corresponds to the molecular population of the higher vibrational energy level *E*_*i*_ in the ground state. *k*_*B*_ is the Boltzmann constant, and *T* is the temperature of the system. The higher the temperature is, the more molecules will be at the higher vibrational energy levels in the ground state, and the fewer molecules will be at the lowest energy level, resulting in exponential dependence of absorption and emission intensities on the temperature.

To evaluate the thermal sensitivity of ICG’s ASF, the variations of absorption and fluorescence spectra of ICG with temperature were measured (Fig. 1f and Fig. S1). As the temperature increases, absorptions at longer wavelengths (910–920 nm) increase, while the principal absorption peak (794 nm) and the blue energy shoulder (720 nm) decrease at the same time. Correspondingly, as the temperature rises from 303–348 K, the ASF spectrum gradually elevates. In contrast, the SF spectrum excited by the 785 nm CW laser diminishes down due to the decreased absorption at 785 nm for the higher temperature. Based on the obtained experimental results, we conclude that the ASF mechanism in ICG is most likely HBA.

### Evaluating the thermal state of subcutaneous tumors during photothermal treatment

**Fig. 2:**
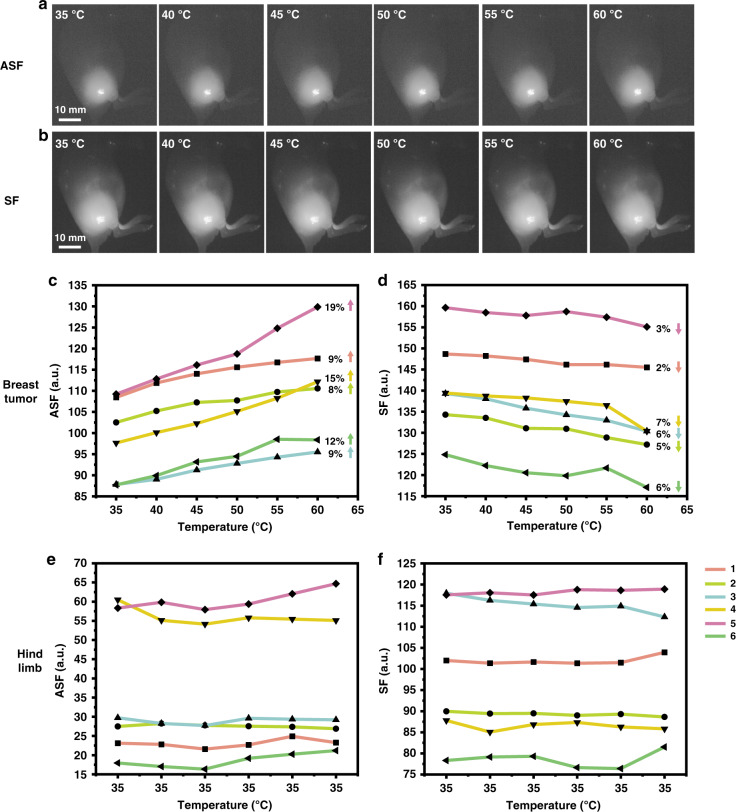
Changes of ICG’s fluorescence intensities in subcutaneous breast tumors of mice during photothermal treatment. Fluorescence images of one tumor mouse reflect the changes of ASF (**a**) and SF (**b**) intensities of ICG in the breast tumor with temperature. Excitation: 915 nm CW laser (40 mW cm^−2^) for ASF, 793 nm laser (5 mW cm^−2^) for SF; fluorescence collection range: 850–900 nm; exposure time: 25 ms for ASF, 1.75 ms for SF. Scale bar: 10 mm. Changes of ASF (**c**) and SF (**d**) intensities of ICG in breast tumors of six mice with temperature. Dynamics of ASF (**e**) and SF (**f**) intensities of ICG in hind limbs of six mice under the condition of constant temperature (35 °C) as controls

We evaluated the capability to use ASF thermal sensitivity to monitor the thermal state of the subcutaneous breast tumors of mice during photothermal treatment using our home-built system (Fig. S2). Photothermal treatment of the breast tumor sites, injected with ICG intratumorally, was performed by irradiation with a 1550 nm laser. This irradiation wavelength was chosen due to high absorption by water in tissues, causing a significant photothermal effect. In contrast, the light of 915 nm or 793 nm wavelength, used to excite the ASF or SF of ICG in the breast tumors, has low absorption by water, and therefore, did not produce any observable thermal effect. Images of tumors at different temperatures controlled by the power of 1550 nm laser were recorded by an imaging camera. Fluorescence images of a representative mouse are displayed in Fig. 2a, b (the other five imaged mice are shown in Fig. S3). The summarized data of 6 mice shows that upon temperature increases, the ASF intensities of ICG in breast tumors significantly increase, while the SF intensities slightly drop (Fig. 2c, d). Taking photobleaching of organic molecules^27,28^ and systematic errors into account, we performed control imaging for which we imaged without 1550 nm laser irradiation, hind limbs of mice intramuscularly injected with ICG. The ASF and SF intensities of ICG in control experiments remain, as expected, almost the same, due to the absence of any temperature change (Fig. 2e, f). ASF of ICG excited by the 915 nm laser is more sensitive to the increase of temperature than SF of ICG excited by the 793 nm laser. ICG-albumin solid samples also present this characteristic. Their ASF intensities are enhanced significantly as temperature increases (from 45–100 °C), which can be used for the high-temperature indication (Fig. S4).

### ASF imaging: ICG vs. UCNP

**Fig. 3:**
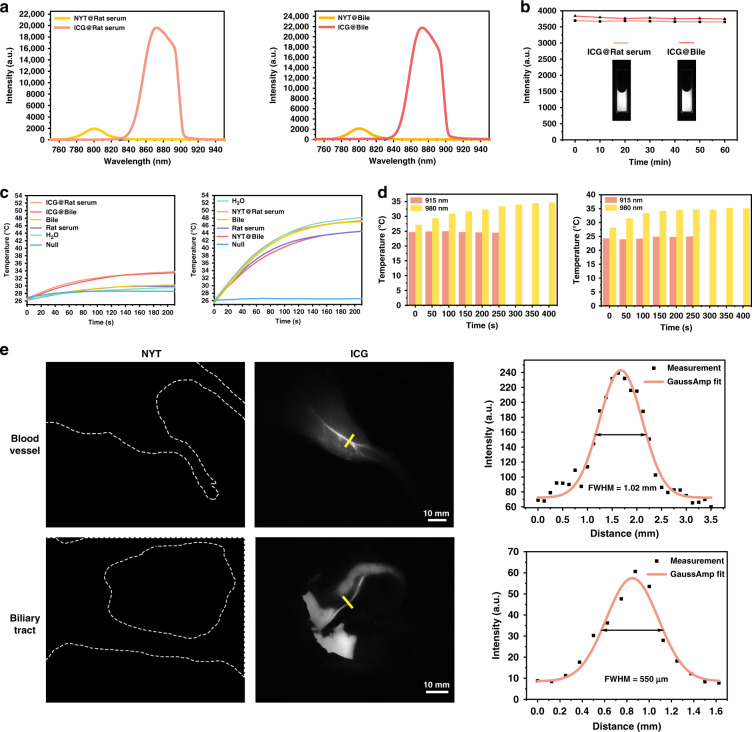
Comparison of ASF performances of ICG and NaYF_4_: Yb^3+^, Tm^3+^ in vitro and in vivo. **a** ASF spectra of ICG (0.1 mg mL^−1^) and NaYF_4_: Yb^3+^, Tm^3+^(1 mg mL^−1^) in rat serum and rat bile. Excitation: 915 nm CW laser (0.9 W cm^−2^) for ICG, 980 nm CW laser (1.8 W cm^−2^) for NaYF_4_: Yb^3+^, Tm^3+^; integration time: 0.5 s for ICG, 4 s for NaYF_4_: Yb^3+^, Tm^3+^. **b** Photostability of ICG (0.1 mg mL^−1^) in rat serum and rat bile under the continuous irradiation of 915 nm CW laser (68 mW cm^−2^) for one hour. Insert: the corresponding ASF images, fluorescence collection range: 800–900 nm, exposure time: 25 ms. **c** In vitro measurements of photothermal effects for simulating the in vivo experimental scenes. ICG Group (left): 915 nm CW laser (1.8 W cm^−2^) irradiation, NaYF_4_: Yb^3+^, Tm^3+^group (right): 980 nm CW laser (1.8 W cm^−2^) irradiation. **d** The photothermal effects on rats’ hind limbs (left) and livers (right) in in vivo wide-field imaging, using ICG excited by the 915 nm CW laser (16.5 mW cm^−2^) or directly irradiating with the 980 nm CW laser (0.6 W cm^−2^). **e** In vivo ASF wide-field imaging of blood vessels and biliary tracts of two rats after receiving an injection of NaYF_4_: Yb^3+^, Tm^3+^and ICG respectively. Imaging conditions: 980 nm CW laser (57 mW cm^−2^) irradiated on the rat injected with NaYF_4_: Yb^3+^, Tm^3+^(9.6 mg mL^−1^, 500 μL); 915 nm CW laser irradiated on the hind limb (16.5 mW cm^−2^) and the biliary tract (4.5 mW cm^−2^) of the rat injected with ICG (1 mg mL^−1^, 500 μL); fluorescence collection range: 800–900 nm; exposure time: 25 ms. The FWHMs of the blood vessel (1.02 mm) and the biliary tract (550 μm) along yellow solid lines were measured. Scale bar: 10 mm. Abbreviations: NYT NaYF_4_: Yb^3+^, Tm^3+^

UCNP is a popular anti-Stokes fluorescent material often used in biological imaging. However, such inorganic nanoparticles usually exhibit some deficiencies, including weak brightness, needed complex surface modification, and long metabolism time in the organism, which limit their applications in vivo to a certain extent^29,30^. ICG is an FDA-approved clinical fluorescent agent, so we chose it for ASF bioimaging and compared it with NIR-I fluorescent UCNP, NaYF_4_: Yb^3+^, Tm^3+^^31^. Comparison on ASF intensity was performed in vitro at first. From the ASF spectra of ICG and NaYF_4_: Yb^3+^, Tm^3+^in rat serum, and rat bile (Fig. 3a), it can be seen that the peak fluorescence intensity of ICG at 865 nm can reach 20,000, while that of NaYF_4_: Yb^3+^, Tm^3+^ at 800 nm is only around 2000 even under the conditions of higher excitation power and longer integration time. ICG possesses much brighter ASF than that of NaYF_4_: Yb^3+^, Tm^3+^, both in rat serum and rat bile. The photobleaching resistance analysis of ICG in rat serum and rat bile were also performed. The 915 nm CW laser continuously irradiated on the samples for one hour with the intensity of 68 mW cm^−2^, which is sufficient for the subsequent wide-field ASF in vivo imaging. ICG has almost no attenuation in ASF intensities (Fig. 3b), suggesting its excellent photostability.

In view of the fact that the optimal excitation wavelength (980 nm) for NaYF_4_: Yb^3+^, Tm^3+^ is at one of water absorption peaks, while the 915 nm excitation for ICG has quite low absorption by water^32^, the photothermal effects caused by these two excitations are likely to be different. The measurement of photothermal effects in vitro was performed at first, using ICG or NaYF_4_: Yb^3+^, Tm^3+^ excited by 915 nm or 980 nm CW laser. In the ICG system, under continuous irradiation from the 915 nm CW laser (1.8 W cm^−2^), the temperature rise of the system (8 °C, from 26–34 °C in the ICG^@^Rat serum/ICG^@^Bile curve) mainly comes from the photothermal effect of ICG itself (4 °C, the highest temperature in the Rat serum/Bile curve is 30 °C), which is not high (Fig. 3c and Fig. S5). Then, the measurement of photothermal effects in vivo wide-field imaging was carried out. The hind limb and the liver of a rat injected with ICG were continuously irradiated with the 915 nm CW laser (16.5 mW cm^−2^) for 250 s, and as expected temperatures remained basically unchanged (Fig. 3d and Fig. S6). On the contrary, in the NaYF_4_: Yb^3+^, Tm^3+^system, under the continuous irradiation of 980 nm CW laser (1.8 W cm^−2^) which could only excite weak signals of NaYF_4_: Yb^3+^, Tm^3+^, the temperature rise of the system (20 °C, from 27–47 °C in the NYT^@^Rat serum curve) mainly comes from the strong water absorption at 980 nm (18 °C, from 26–44 °C in the Rat serum curve), and the photothermal effect is more serious than that in the ICG system (Fig. 3c and Fig. S5). The hind limb and the liver of another rat without the injection of NaYF_4_: Yb^3+^, Tm^3+^ (the absorption of NaYF_4_: Yb^3+^, Tm^3+^ at 980 nm is negligible, but this group is still defined as “NaYF_4_: Yb^3+^, Tm^3+^ group”) were continuously irradiated with the 980 nm CW laser (0.6 W cm^−2^) for 400 s till temperatures no longer changed, and temperatures of them both increase by ~7 °C (Fig. 3d and Fig. S6). During the study of photothermal effects on rats treated with ICG and NaYF_4_: Yb^3+^, Tm^3+^ groups, the intensities of 915 nm (16.5 mW cm^−2^) and 980 nm (0.6 W cm^−2^) CW lasers were set on the premise that the fluorescence intensities of ICG and NaYF_4_: Yb^3+^, Tm^3+^ were close. So, another advantage of using ICG for in vivo ASF imaging compared with NaYF_4_: Yb^3+^, Tm^3+^ is that it can effectively avoid photothermal damage to biological tissues.

In vivo wide-field ASF imaging of rats was further conducted using ICG and NaYF_4_: Yb^3+^, Tm^3+^. A blood vessel in the hind limb, as well as the biliary tract of a rat intravenously injected with ICG, can be clearly identified. The full widths at half maxima (FWHMs) of the imaged blood vessel and biliary tract were measured as 1.02 mm and 550 μm respectively. Standard deviations of measured FWHMs are 0.064 mm and 9.88 μm for the blood vessel and the biliary tract respectively, and the corresponding coefficients of variation are 6.3% and 1.8% respectively (Fig. S7). In contrast, no fluorescence signal could be detected in blood vessels and the biliary tract of the rat intravenously injected with NaYF_4_: Yb^3+^, Tm^3+^ (Fig. 3e).

### ICG’s ASF for micro-angiography and organ multi-mode imaging in vivo

#### In vivo intra-vessel blood stream monitoring

**Fig. 4:**
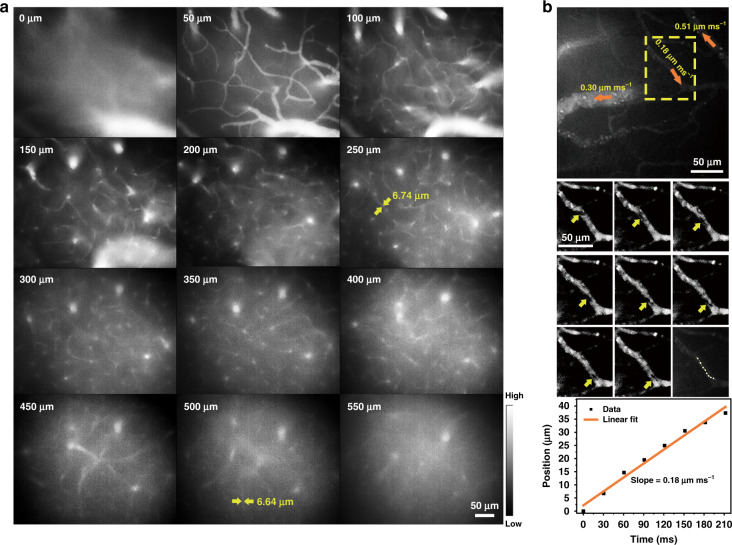
The application of ICG’ASF in cerebral vascular tomography and flow velocity measurement. **a** In vivo ASF wide-field microscopic images of brain blood vessels (at various depths from 0 μm to 550 μm) of the mouse injected with ICG (5 mg mL^−1^, 250 μL). Excitation: 915 nm CW laser; fluorescence collection range: 800–900 nm; exposure time: 25 ms. Scale bar: 50 μm. **b** Flow velocity measurements of three sampled brain blood vessels. Orange arrows indicate the directions of blood flow. Frames of the yellow dashed box were recorded (middle in the right), showing the tracking of a dark spot in one cerebral vessel. A fitting line (bottom in the right) revealing the position of the spot as a function of time is shown. The slope of the fitting line stands for the blood flow velocity (0.18 μm ms^−1^) in this cerebral vessel. Excitation: 915 nm CW laser; fluorescence collection range: 800–900 nm; exposure time: 30 ms. Scale bar: 50 μm

Benefiting from ICG’s bright NIR-I ASF, ICG was used for tomography of the cerebral vessels in the mouse with our home-built wide-field ASF microscope (Fig. S8). After being implanted with a cranial window under anesthesia, the mouse was intravenously injected with ICG and its cerebral vessels were imaged. The imaging depth is 550 μm and the spatial resolution at the depth of 500 μm can reach 6.64 μm (Fig. 4a). Under some basic conditions such as anesthesia, the flow velocity in a blood vessel should be constant. Thus, the cerebrovascular flow velocity was also measured. Based on a ~25× objective, some dark spots (blood cells) without ICG flowing along blood vessels could be observed (Supplementary video MOV S1 online). In order to calculate cerebrovascular flow velocity, a dark spot in a brain blood vessel pointed by the yellow arrow in the yellow dashed box was selected, and its position was continuously tracked at 33 frames per second. By positioning this spot in different frames in the same field of view, a linear fitting for the position-time relationship of the spot was performed, and the slope, i.e. the cerebrovascular flow velocity of this vessel, is 0.18 μm ms^−1^ (Fig. 4b). In the same way, the flow velocities of the other two brain blood vessels were calculated as 0.30 μm ms^−1^ and 0.51 μm ms^−1^.

#### ICG’s ASF for multi-mode imaging of organs

Multi-mode imaging, wherein a combination of various properties in one or several probes is used, can provide more detailed information about the investigated biological sample than traditional imaging methods^33^.

**Fig. 5:**
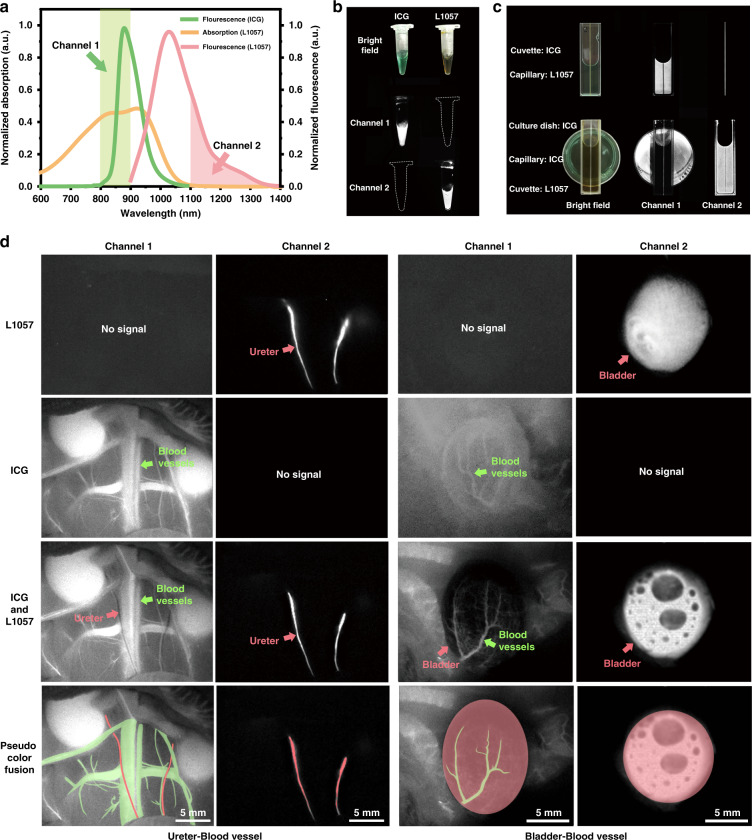
Multi-mode imaging of urinary system and blood vessels under the excitation of a single light source. **a** The fluorescence spectra of ICG in rat serum and L1057 NPs in water, and the absorption spectrum of L1057 NPs in water. **b** Bright-field images and fluorescence images of ICG (0.1 mg mL^−1^ in rat serum) and L1057 NPs (0.5 mg mL^−1^ in water) in channel 1 and channel 2 respectively. Excitation: 915 nm CW laser, 13 mW cm^−2^; exposure time: 25 ms for channel 1, 11 ms for channel 2. **c** In vitro simulation of multi-mode imaging. The upper part simulates the ureter-blood vessel scenario, where the capillary filled with L1057 NPs simulates the ureter, and the cuvette filled with ICG simulates underlying blood vessels and tissues. The lower part simulates the bladder-blood vessel scenario, where the capillary filled with ICG simulates the vessel on the bladder surface, the cuvette filled with L1057 NPs simulates the bladder, and the culture dish filled with ICG simulates the underlying tissues. Excitation: 915 nm CW laser, 13 mW cm^−2^; exposure time: 25 ms for channel 1,15 ms for channel 2; dosage: 0.1 mg mL^−1^ of ICG in rat serum, 0.5 mg mL^−1^ of L1057 NPs in water. **d** Multi-mode imaging of rats treated with ICG and L1057 NPs under the excitation of a 915 nm CW laser. In channel 1: excitation: 15 mW cm^−2^; exposure time: 25 ms. In channel 2: excitation: 55 mW cm^−2^; exposure time: 30 ms. Dosage: ICG (2 mg mL^−1^, 300 μL), L1057 NPs (0.15 mg mL^−1^, 1 mL). Scale bar: 5 mm

Combining ICG with L1057 nanoparticles (NPs), which is a NIR fluorescent organic polymer dot^34^, we bridged two modes, ASF and traditional SF imaging, to produce a multi-mode imaging technique, providing image separation of two organs with enhanced contrast. In our multi-mode imaging scheme, we used a single light source for excitation at 915 nm (Fig. 5a). As we described above, this wavelength efficiently excites ASF of ICG in the 800–900 nm spectral window, which we used as the first imaging channel (Channel 1, Fig. 5a). At the same time, if ICG and L1057 NPs are co-localized or nearly localized, the generated ASF of ICG will be efficiently absorbed by L1057 NPs due to the strong absorption of L1057 NP in this spectral range (Fig. 5a). Because anti-Stokes excitation is not efficient compared to Stokes one, generated ASF of ICG can be fully absorbed by L1057 NP, producing a negative contrast in channel 1. Meanwhile, the 915 nm excitation can efficiently excite L1057 NPs, emitting SF in the 1100–1400 nm spectral window, which we used as the second imaging channel (Channel 2, Fig. 5a). Using our home-built system (Fig. S9), this idea of multi-mode imaging with single excitation was tested in vitro at first. Two different imaging channels show images of two different probes, which can model the distribution of probes in two different organs in a live organism (Fig. 5b). A negative contrast is demonstrated in channel 1 by the capillary containing L1057 NPs, embedded in a cuvette with the ICG solution. In channel 2, only the capillary is positively visualized (Fig. 5c). In this in vitro experiment, the capillary is used as a biological ureter model.

For in vivo experiments, ICG was intravenously injected in rats while L1057 NPs were retrogradely injected into the ureters through urethras (Fig. S10). According to these procedures, ICG is distributed only in the blood vessels of the rat, while L1057 NPs are distributed only in ureters. As shown in the left part of Fig. 5d, when just ICG was injected, only channel 1 shows fluorescence signals. Blood vessels and some other organs (e.g. kidneys) are bright, accompanied by the scattered fluorescence in surrounding tissues, while ureters could not be visualized at all. When just L1057 NPs were injected, only channel 2 shows fluorescence signals visualizing ureters. When both ICG and L1057 NPs were injected, the channel 1 image clearly shows ASF of ICG in blood vessels, while in ureters the ASF of ICG is completely absorbed by the L1057 NPs inside, producing dark visualization of this organ. At the same time, absorption of ASF of ICG in blood vessels by L1057 NPs is not so intense due to a larger spatial separation of two probes, giving enough intensity in channel 1 for blood vessels imaging. In channel 2, only ureters are visualized without crosstalk of fluorescence signals in blood vessels.

Another impressive example of multi-mode imaging application is shown in Fig. 5d, right two columns, when ICG intravenously and L1057 NPs retrogradely to bladder were injected. Bright imaging of blood vessels (ICG’s ASF) are shown on the dark background of the bladder (L1057 NPs’ absorption, i.e. negative imaging), providing an excellent contrast of imaging in channel 1, while in channel 2 detailed imaging of bladder (L1057 NPs’ fluorescence) is exclusively shown. It is worth noting that the blood vessels on the bladder are much clearer in the image, compared to that in the channel 1 image, obtained without L1057 NPs. The video of multi-mode imaging can be seen in Supplementary video MOV S2 online.

Interestingly, since ICG can produce fluorescence signals above 1100 nm (Fig. S11), when ICG is excited by the 793 nm laser (strongly absorbed by ICG), the SF of ICG in channel 2 is very strong and has serious crosstalk with that of L1057 NPs (which can also be effectively excited by 793 nm laser), rendering the blood vessels and ureters all bright and they could not be distinguished at all (Fig. S12). The results illustrate that together with other NIR fluorescent probes and under a single light source (wavelength near 793 nm) excitation, the SF of ICG will interfere with the positive imaging of the organ labeled by other NIR fluorescent probes.

The residue of L1057 NPs in the urinary system of the rat was also analyzed. 48 h after the retrograde injection of L1057 NPs, there was no fluorescence signal in the urinary system (Fig. S13a). The fluorescence signal of the urine from the rat, which was collected from 2–48 h after the rat being injected with L1057 NPs, gradually weakened and finally disappeared (Fig. S13b). The stability of L1057 NPs in urine was also studied. It can be seen that the absorption and fluorescence intensity of L1057 NPs hardly changed after being dissolved in the urine for 48 h (Fig. S13c and Fig. S13d). Thus, the reduction of fluorescence of L1057 NPs in urine as time went by should not be attributed to their instability, indicating L1057 NPs can be completely excreted from the urinary system.

## Discussion

In summary, we found that ICG could generate bright NIR ASF due to HBA under the excitation of CW laser at 915 nm. This ASF intensity is enhanced as temperature rises, and thus was utilized for monitoring the thermal state of subcutaneous tumors during photothermal treatments. Besides, the ASF of the certain ICG sample can be also used for the high-temperature indication (more suitable than its SF). Benefiting from its FDA-approved status, high emission efficiency even in the anti-Stokes mode of excitation, together with favorable excitation wavelength avoiding photothermal damage to biological tissues, and resistance to photobleaching, ICG shows promising applications in in vivo bioimaging fields, such as deep tomography of cerebral blood vessels and measurement of the blood flow velocity of the mouse. Furthermore, combining ICG with organic polymer dots, L1057 NPs, we achieved real-time advanced multi-mode bioimaging in vivo under a single CW light source excitation at 915 nm. This modality is very promising for intraoperative visualization which may help avoid accidental injury to adjacent blood vessels during clinical operations, and it can also be used for other clinical imaging scenes (e.g. identify lymphatic system and urinary system simultaneously). Interestingly, ICG can generate visible ASF ( <700 nm) under 915 nm CW laser excitation (Fig. S11 and Fig. S14), which may help human being perceive infrared light by naked eyes. Followed by our work, other fluorophores with HBA-induced ASF features can be synthesized and utilized for bioimaging, sensing, theranostics, and infrared light perception in the future.

## Materials and methods

### Materials

ICG was purchased from DanDong Pharmaceutical Factory (Liaoning, China). DMSO was purchased from Sinopharm Chemical Reagent Co., Ltd., China. The UCNPs (NaYF_4_: Yb^3+^, Tm^3+^) were synthesized according to our previous report^35^. Fresh rat serum and bile were obtained from rats in our laboratory. L1057 NPs were provided by Prof. Jie Liu’s group in Nanjing Tech University. Phosphate buffered saline (PBS) was purchased from Sinopharm Chemical Reagent Co., Ltd., China.

### Experimental setup for measuring absorption spectra, fluorescence spectra, and power dependence (915 nm CW laser excitation)

The absorption spectra of ICG and L1057 NPs were measured by UV-VIS-NIR spectrophotometer (CARY 5000, Agilent). The fluorescence spectra of ICG, NaYF_4_: Yb^3+^, Tm^3+^, and L1057 NPs were acquired on a home-built system based on the PG2000 spectrometer (370–1050 nm, Ideaoptics Instruments) and NIR2200 spectrometer (900–2200 nm, Ideaoptics Instruments). The dependence of the ASF intensity of ICG in DMSO on the intensity of 915 nm CW laser excitation was derived from ICG’s fluorescence spectra excited at different powers.

### Experimental setup for measuring lifetime and power dependence (915 nm fs pulsed laser excitation)

The fs pulsed laser (PHAROS PH1-10W, LIGHT CONVERSION; repetition rate: 1 MHz; pulse width: 200 fs) beam was introduced into a commercial inverted microscope (IX83, Olympus, Japan) as the excitation light. After being reflected by the dichroic mirror and passing through the objective (PLN20X, Olympus), the laser beam excited the sample. Based on a turnover mirror, emitted fluorescence signals passing through the dichroic mirror and filters were either delivered to a spectrometer (Andor, 193i + iXon DU-897U) or detected by an avalanche photodiode (τ-SPAD, PICOQUANT). The computer with an integrated TCSPC module system (DPC-230 Photon Correlator, Becker & Hickl GmbH) was used to measure the fluorescence lifetime of the sample based on the synchronous signals output by the fs laser and electrical signals from the τ-SPAD. The dependence of the ASF intensity of ICG in DMSO on the intensity of 915 nm fs pulsed laser excitation was derived from ICG’s fluorescence spectra excited at different powers.

### Cell lines and cell culture

Mouse breast cancer cell line (4T1) was purchased from the Type Culture Collection of the Chinese Academy of Sciences (Shanghai, China). 4T1 cell line was cultured in RPMI-1640 (Gibco, Cat. No. C11975500BT) supplemented with 10% fetal bovine serum (FBS, Cellmax, Cat. No. SA102.02) and maintained at 37 °C with 5% CO_2_.

### Animal preparation

BALB/c nude mice (female, 6 weeks old), Sprague Dawley rats (female, 180 g), and Institute of Cancer Research (ICR) mice (female, 6 weeks old) were used for in vivo experiments. They were provided by the Zhejiang Academy of Medical Sciences and kept at the Experimental Animal Center of Zhejiang University. The room temperature of the rearing environment was maintained at 24 °C with a 12 h light/dark cycle. Rats and mice were continuously supplied with water and standard laboratory chows. All in vivo experiments were approved by the Institutional Ethical Committees of Animal Experimentation of Zhejiang University (ZJU20190076) and Sir Run-Run Shaw Hospital Affiliated to the School of Medicine in Zhejiang University (SRRSH2021401), and strictly abided by “The National Regulation of China for Care and Use of Laboratory Animals”. In the experiment of evaluating the thermal state of subcutaneous tumors during photothermal treatment, BALB/c nude female mice were subcutaneously injected with 4T1 cells (5 × 10^5^) in 150 μL of PBS. Mice were observed till their tumors grew to 5 mm in diameter. One day before the observation, their hind limbs were intramuscularly injected with ICG, and 1 h before the observation, they were intratumorally injected with ICG. In whole-body imaging experiments, rats were anesthetized at first, and they were fixed on the imaging platform in the supine position. Shaving and laparotomy were performed to completely expose the imaging area of interest. Finally, rats were injected with fluorescent probes. Among them, ICG assisted angiography is special. Since ICG metabolizes fast in blood vessels, an intravenous indwelling needle was placed in the vein of the rat to supply ICG when needed. In the wide-field microscopic cerebrovascular imaging experiment, the skull of the anesthetized ICR mouse was opened by microsurgery. Then a thin round cover glass with double wings was attached to the mouse brain by dental cement. The purpose of doing this is to protect and flatten the brain of the mouse to ensure the quality of microscopic imaging. Before imaging, both wings were fixed on the mouse rack to immobilize mouse’s head, and it was then intravenously injected with ICG (5 mg mL^−1^, 250 μL).

### Experimental setup for evaluating the thermal state of subcutaneous tumors during photothermal treatment

This system is divided into two parts. One part is used for fluorescence imaging and the other part is used for photothermal treatment. For imaging, 915 nm CW laser beam for ASF excitation and 793 nm CW laser beam for SF excitation were separately coupled to two collimators through two optical fibers. The 793 nm laser beam was reflected by an 805 nm long-pass dichroic mirror (DMLP805R, Thorlabs) while the 915 nm laser beam was transmitted through the dichroic mirror. Next, either beam could be expanded by a lens with a ground glass sheet, which was used to eliminate laser speckles and provide a large illumination area. Fluorescence signals from tumors of mice were captured by a wide spectral responsive Si-based camera (GA1280, 1280 pixels × 1024 pixels, TEKWIN SYSTEM, China) after passing through the prime lens (focal length: 35 mm) with an antireflection coating at 800–1700 nm and a combination of a 900 nm short-pass filter (FESH0900, Thorlabs) and an 850 nm long-pass filter (FEL0850, Thorlabs) filtering away the excitation light. For photothermal treatment, 1550 nm CW laser beam was coupled to a collimator (F810FC-1550, Thorlabs) through the optical fiber, and then the collimated laser beam irradiated on the subcutaneous breast tumor of the mouse. A thermal imager (TiS20, FLUKE) was used to record the precise temperature in photothermal treatment.

### Wide-field microscopy for cerebrovascular imaging

A home-built system was established to perform ASF wide-field microscopic imaging of cerebral vessels in mice. 915 nm CW laser beam as the excitation light source passing a 900 nm long-pass filter (FELH0900, Thorlabs) was incident on a 900 nm short-pass dichroic mirror (#69-222, Edmund Optics) and then reflected, irradiating onto the mouse brain through an infrared antireflection water immersion objective (XLPLN25XWMP2, 25×, NA = 1.05, Olympus). The excited ASF was collected by the same objective, and then passed through the same 900 nm short-pass dichroic mirror and a combination of a 900 nm short-pass filter (FESH0900, Thorlabs) and an 800 nm long-pass filter (FELH0800, Thorlabs). Finally, ASF signals were focused on a wide spectral responsive Si-based camera (GA1280, 1280 pixels × 1024 pixels, TEKWIN SYSTEM, China) through the built-in tube lens in the trinocular to visualize the cerebral vessels of the mouse. The system was equipped with an electric control module, which could control the objective (together with the whole microscope unit) to move along the Z-axis direction and collect the signals at different depths of the mouse brain for depth tomography. It could also control the loading platform to move in the X-Y direction and change the imaging field of view.

### Multi-mode imaging setup for simultaneous visualization of the urinary system and blood vessels

The 915 nm CW laser beam was coupled to a collimator through the optical fiber, and then expanded by a lens with a ground glass sheet which could eliminate laser speckles to provide a large illumination area. There were two sets of imaging systems placed parallel to each other, and one was the ASF imaging channel. Fluorescence signals were captured by a wide spectral responsive Si-based camera (GA1280, 1280 pixels × 1024 pixels, TEKWIN SYSTEM, China) after passing through the prime lens (focal length: 35 mm) with an antireflection coating at 800–1700 nm and a combination of a 900 nm short-pass filter (FESH0900, Thorlabs) and an 800 nm long-pass filter (FELH0800, Thorlabs) filtering away the excitation light. The other was the SF imaging channel, which consisted of the same prime lens, a 1100 nm long-pass filter (FELH1100, Thorlabs), and an InGaAs camera (640 pixels × 512 pixels, TEKWIN SYSTEM, China). The two sets were next to each other, and the rat was placed in their common field of view.

## Supplementary information

Supplementary information

Supplementary video MOV S1

Supplementary video MOV S2
