# Supplementary material for: Hot-band absorption of indocyanine green for advanced anti-stokes fluorescence bioimaging
Source: Light Sci Appl. 2021 Sep 13;10:182. doi: 10.1038/s41377-021-00627-1 (PMC8438065; doi:10.1038/s41377-021-00627-1)
Supplement: Supplementary file 1 — Supplementary information [file 41377_2021_627_MOESM1_ESM.docx]

**Supplementary Information for**

Hot-band absorption of Indocyanine Green for advanced anti-Stokes fluorescence bioimaging

Jing Zhou ^1^, Xiaoxiao Fan ^1^, Di Wu ^3^, Jie Liu ^4^, Yuhuang Zhang ^1^, Zikang Ye ^5^, Dingwei Xue ^3^, Mubin He ^1^, Liang Zhu ^6^, Zhe Feng ^1^, Andrey N. Kuzmin ^2^, Wen Liu ^7^, Paras N. Prasad ^2,^*, Jun Qian ^1,^*

^1^ *State Key Laboratory of Modern Optical Instrumentations, Centre for Optical and Electromagnetic Research, College of Optical Science and Engineering, International Research Center for Advanced Photonics, Zhejiang University, Hangzhou 310058, China*

^2^ *Institute for Lasers, Photonics, and Biophotonics, Department of Chemistry, University at Buffalo, State University of New York, Buffalo, NY 14260, USA*

^3^ *Sir Run-Run Shaw Hospital, School of Medicine, Zhejiang University, Hangzhou 310016, China*

^4^ *Key Laboratory of Flexible Electronics (KLOFE) Institute of Advanced Materials (IAM), Nanjing Tech University (Nanjing Tech), Nanjing 211800, China*

^5^ *Department of Chemistry, Zhejiang University, Hangzhou 310058, China*

^6^ *Interdisciplinary Institute of Neuroscience and Technology (ZIINT), College of Biomedical Engineering and Instrument Science, Zhejiang University, Hangzhou 310027, China*

^7^ *Key Laboratory of Optical Information Detection and Display Technology of Zhejiang, Zhejiang Normal University, Jinhua 321004, China*

* Corresponding authors.

*E-mail address:*

Jun Qian [(qianjun@zju.edu.cn](mailto:(qianjun@zju.edu.cn))

Paras N. Prasad [(pnprasad@buffalo.edu)](mailto:(pnprasad@buffalo.edu))


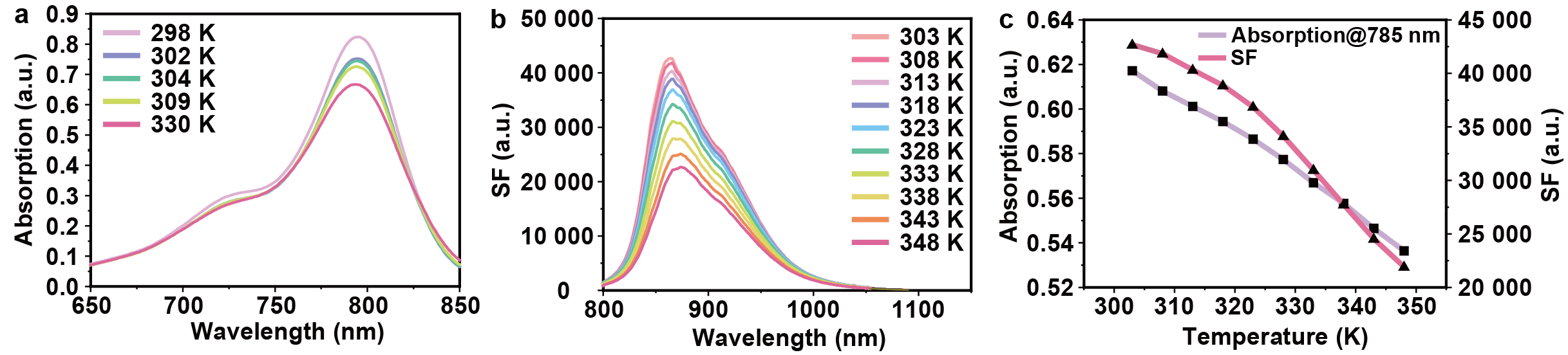


**Fig. S1**. (a) Temperature dependence of ICG’s absorption spectra (650 nm - 850 nm). (b) Temperature dependence of ICG’s fluorescence spectra under the excitation of 785 nm CW laser. (c) Temperature dependence of ICG’s absorption at 785 nm, as well as SF intensity excited by the CW laser at this wavelength.


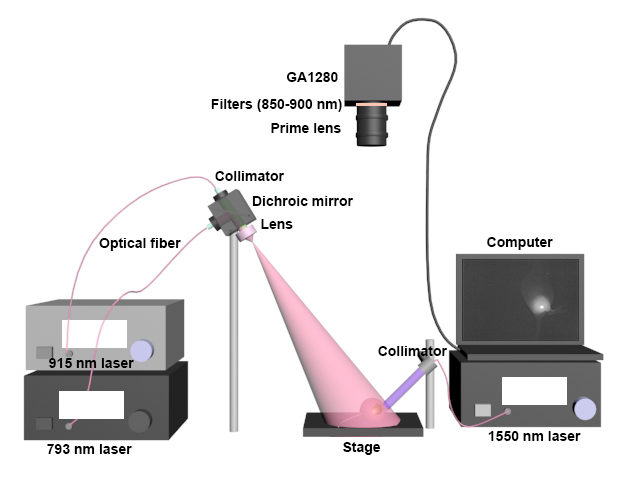


**Fig. S2**. Schematic illustration of the system for evaluating the thermal state of subcutaneous tumors during photothermal treatment.


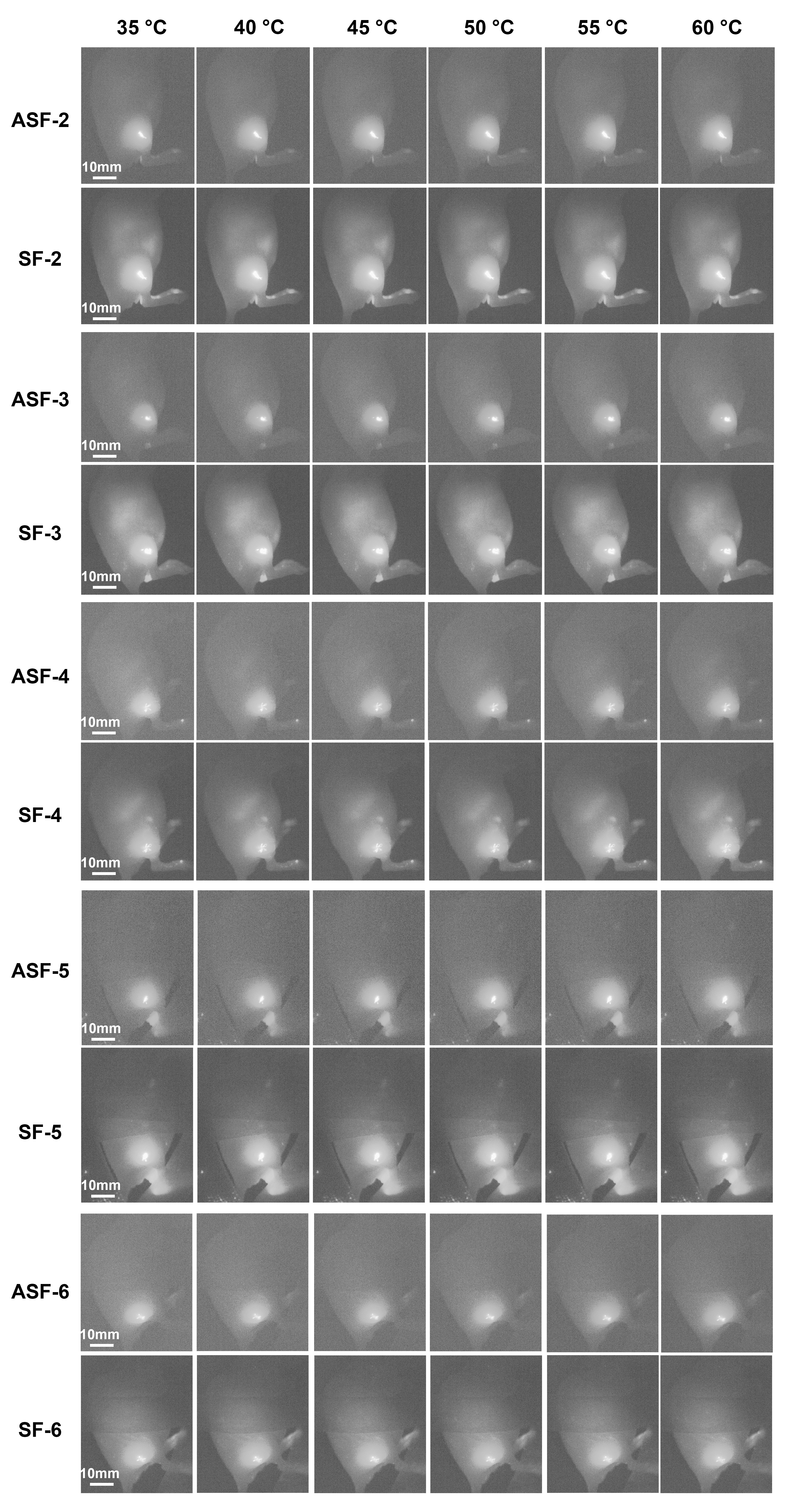


**Fig. S3**. Fluorescence images of the other five tumor mice which reflect the changes of ASF and SF intensity of ICG in the breast tumor with temperature.


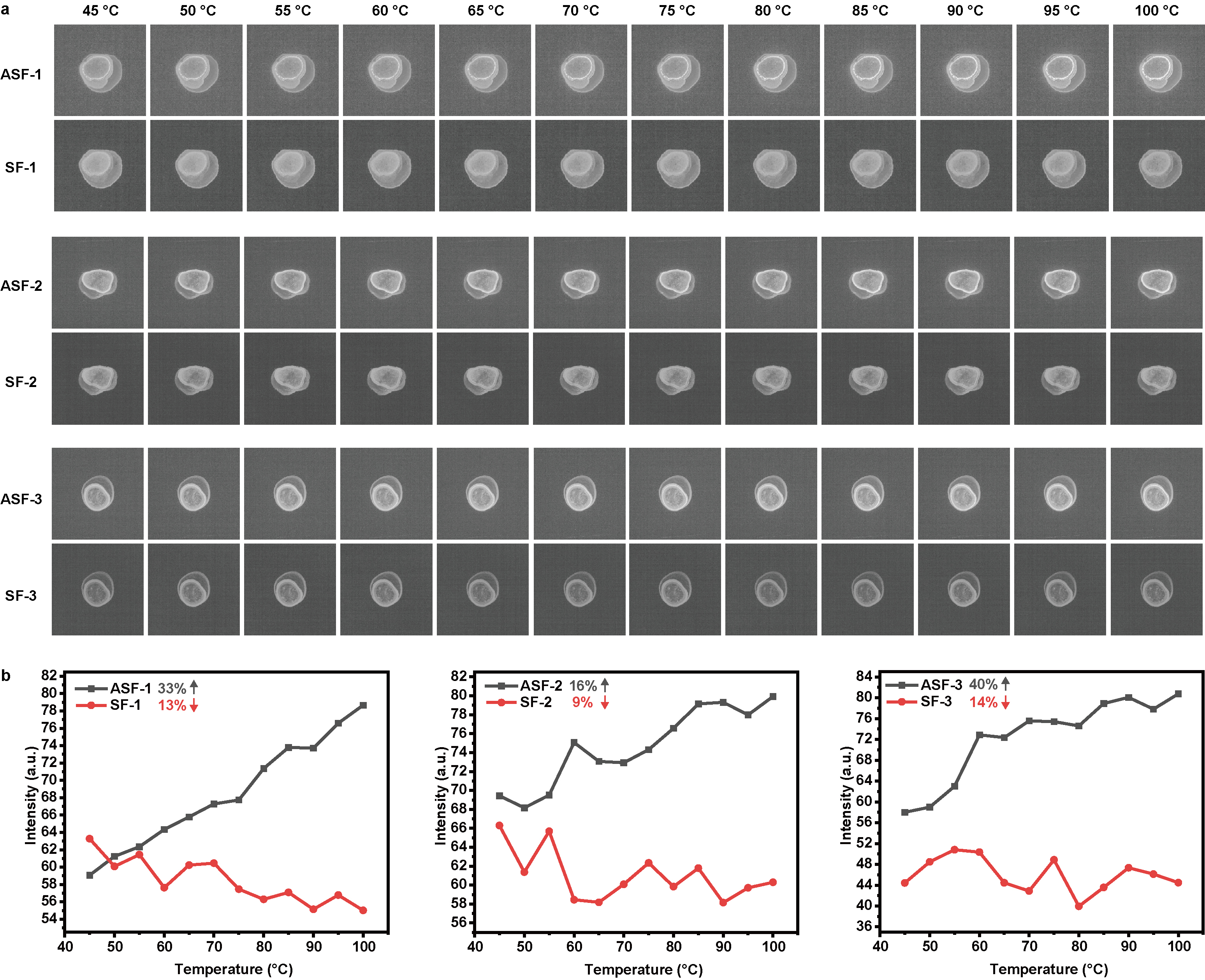


**Fig. S4**. **ASF of ICG for high temperature indication.** (a) Changes in ASF (915 nm laser excited) and SF (793 nm laser excited) images with temperature of three ICG-albumin solid samples. (b) Analyses of intensity changes of ASF and SF of three ICG-albumin solid samples in (a) with temperature.


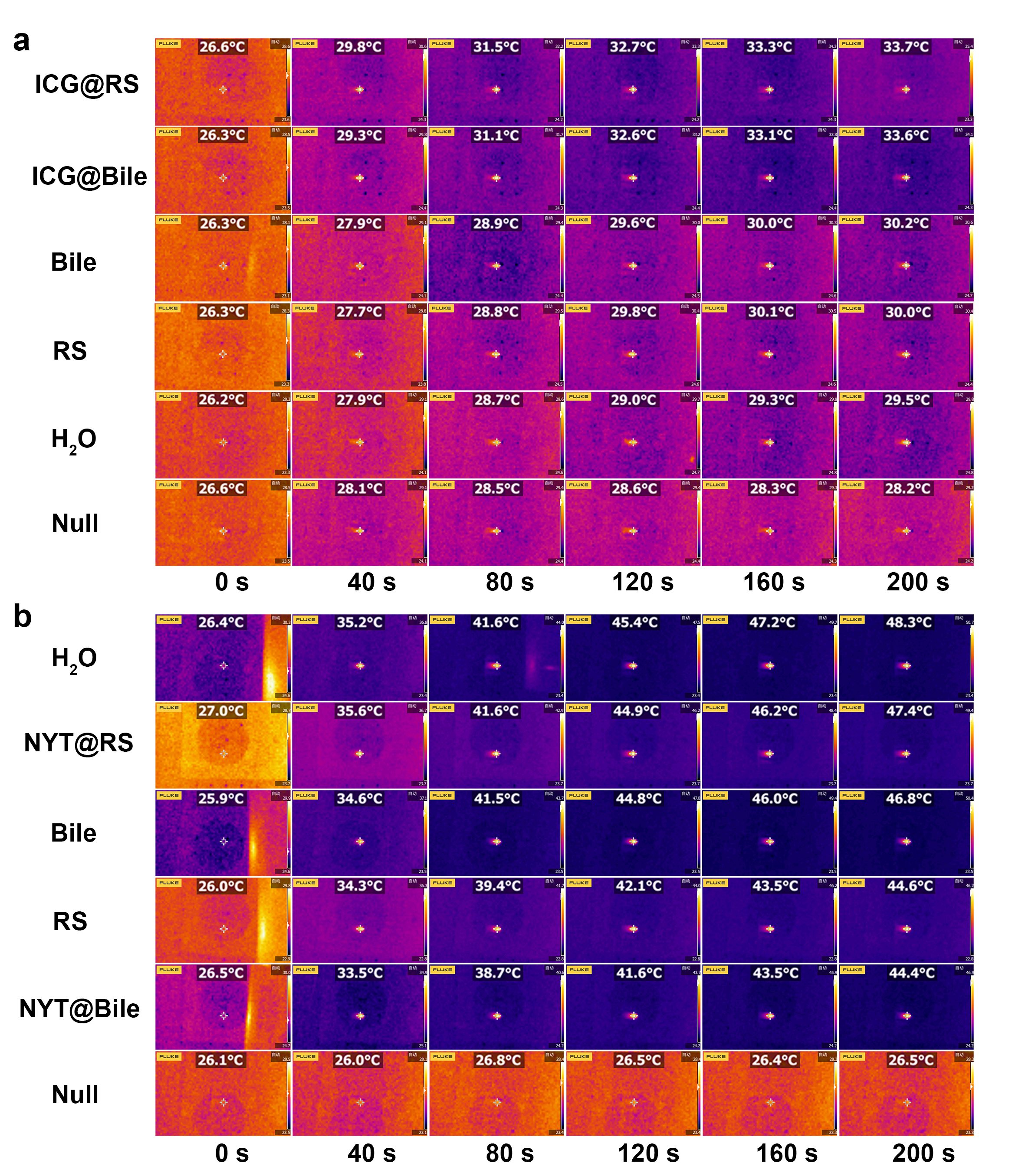


**Fig. S5**. In vitro measurement of photothermal effects with thermal imaging, simulating the in vivo experimental scenes. ICG and NaYF_4_: Yb^3+^, Tm^3+^ systems were excited by (a) 915 nm (1.8 W cm^-2^) and (b) 980 nm (1.8 W cm^-2^) CW lasers respectively. Abbreviations: RS-rat serum; NYT-NaYF_4_: Yb^3+^, Tm^3+^.


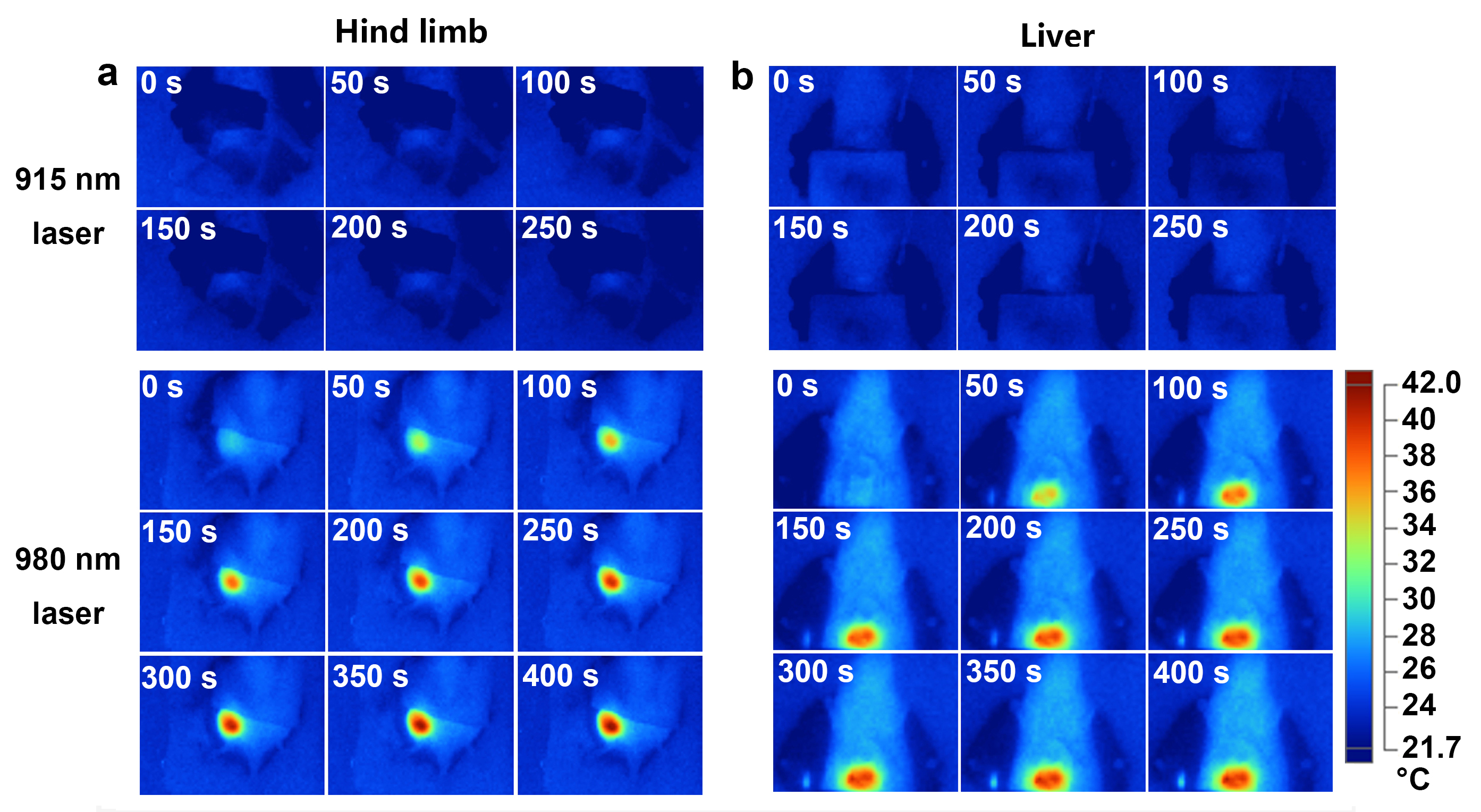


**Fig. S6**. Thermal images of rats’ hind limbs (a) and livers (b) continuously irradiated by the 915 nm laser (16.5 mW cm^-2^) for 250 s or the 980 nm laser (0.6 W cm^-2^) for 400 s.


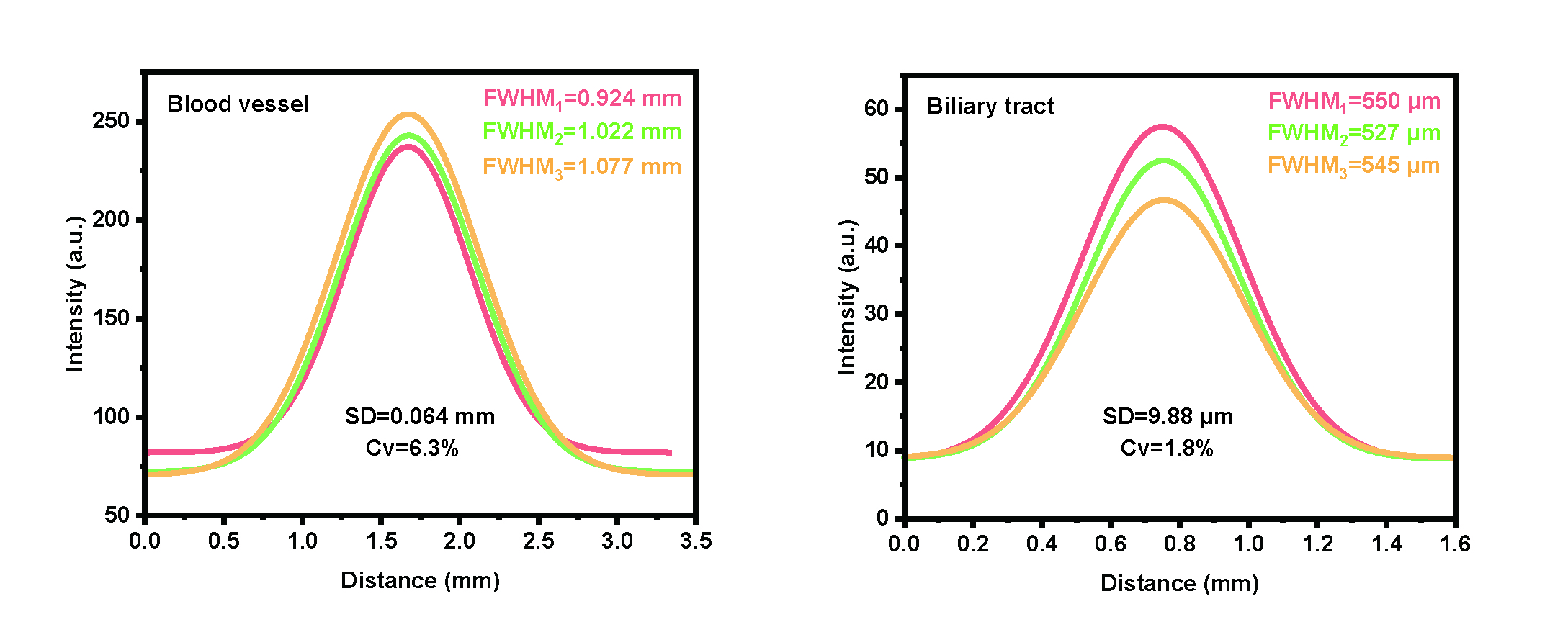


**Fig. S7**. Standard deviations (SD) and coefficients of variation (Cv) of measured FWHMs of the blood vessel and biliary tract.


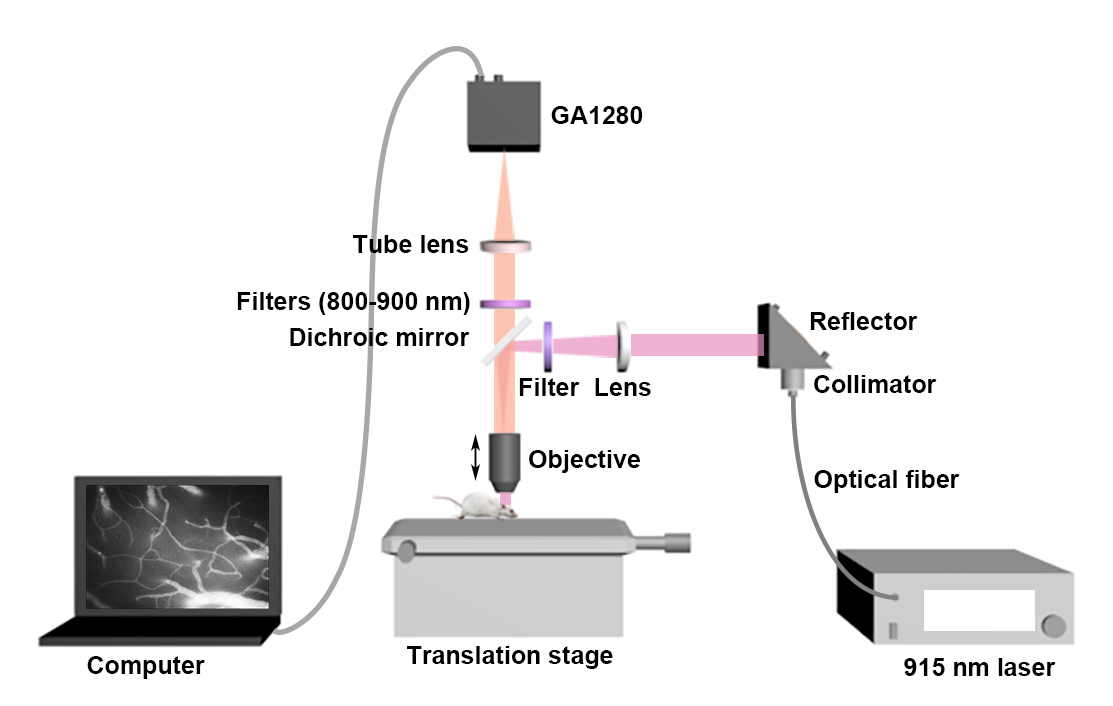


**Fig. S8**. Schematic illustration of the wide-field ASF microscope.


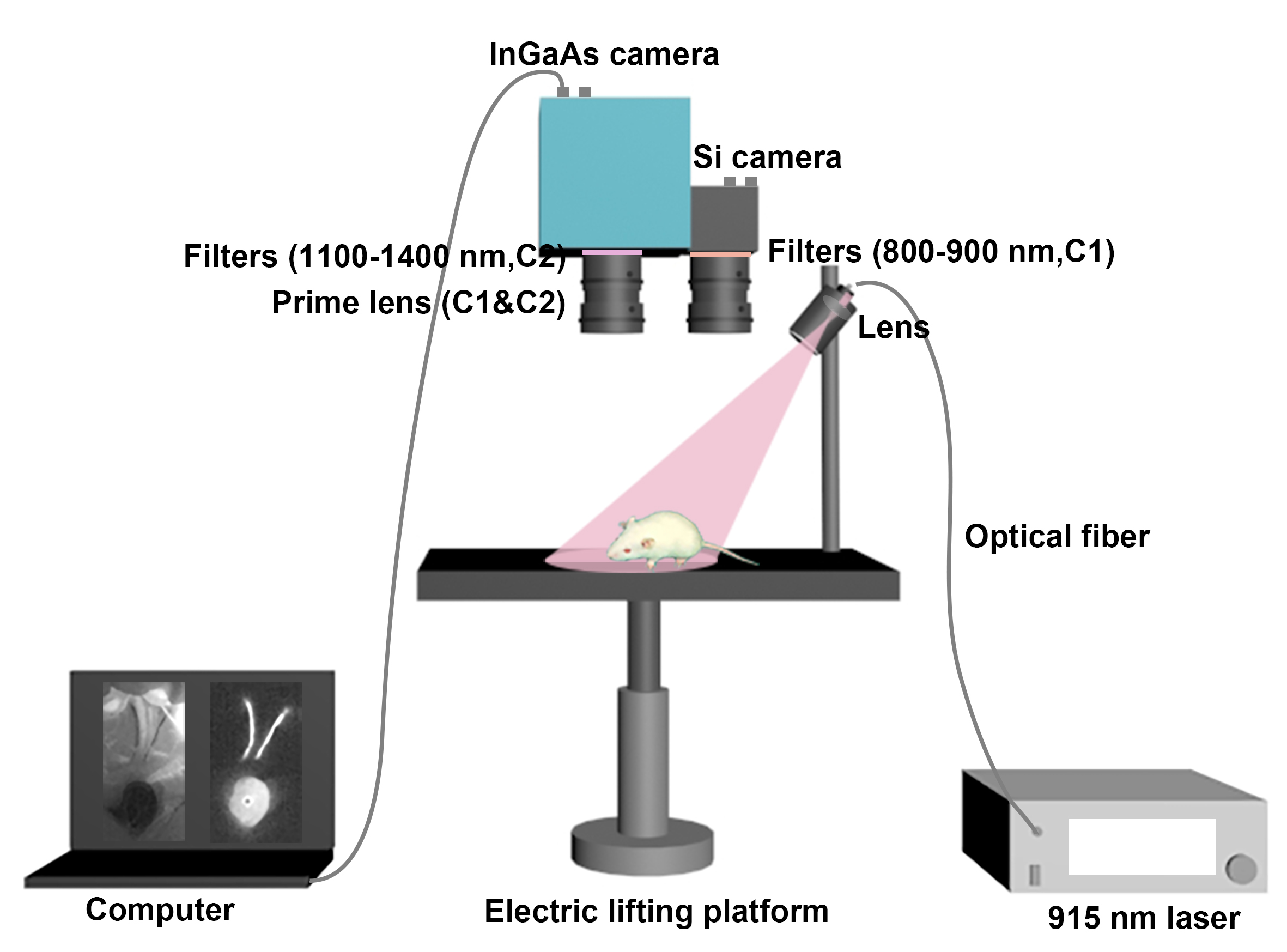


**Fig. S9**. Schematic illustration of the wide-field multi-mode imaging system. Abbreviations: C1-channel 1; C2-channel 2.


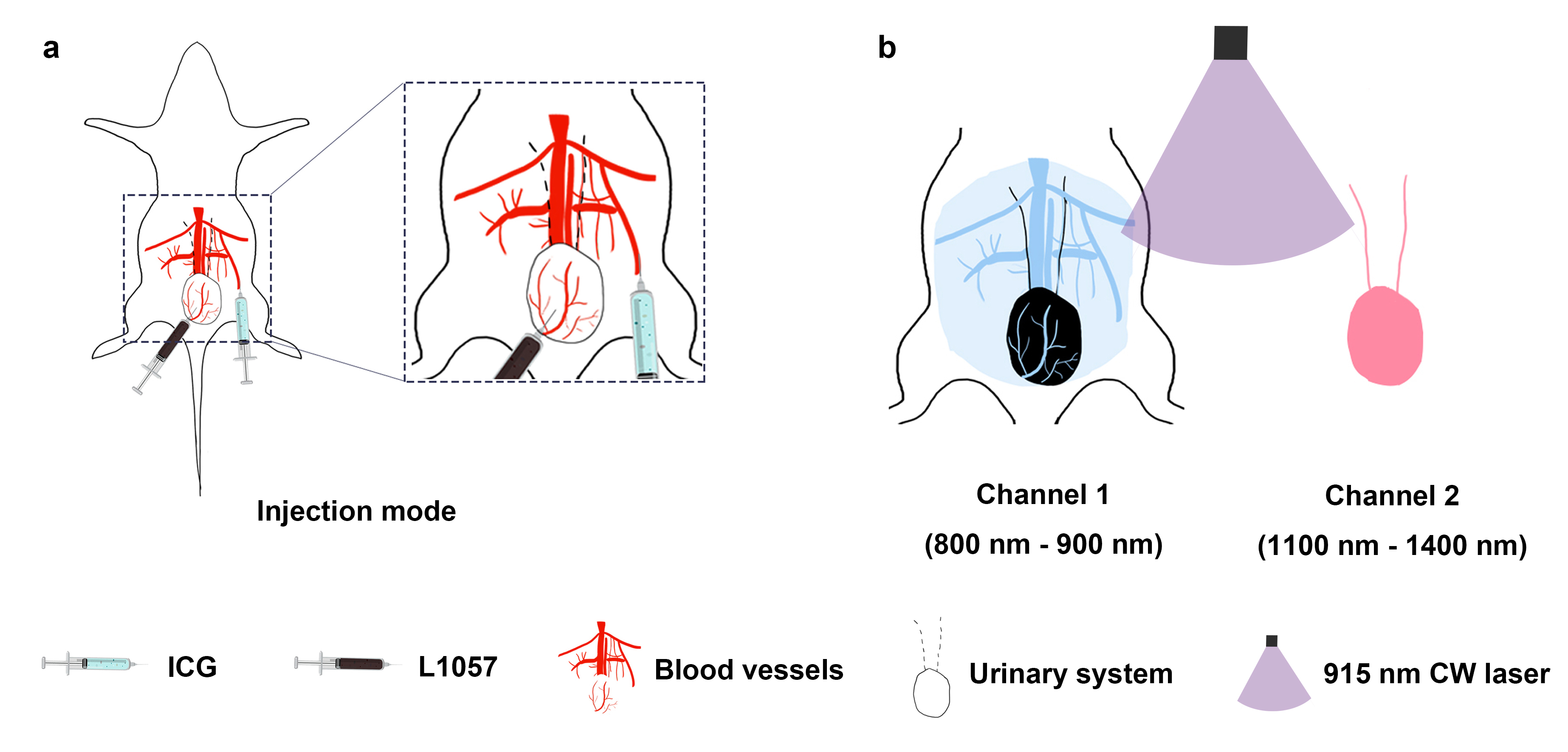


**Fig. S10**. **Schematic illustration of multi-mode imaging under the excitation of a single light source.** (a) ICG is injected into the blood vessels and L1057 NPs are injected into the urinary system. (b) In channel 1 (800 nm – 900 nm), the blood vessels filled with ICG show positive visualization (blue) while the urinary system filled with L1057 NPs shows negative visualization (dark), thus achieving the automatic fusion visualization of blood vessels and urinary system. In channel 2 (1100 nm - 1400 nm), only the urinary system filled with L1057 NPs shows positive visualization (pink), achieving the single-target visualization.


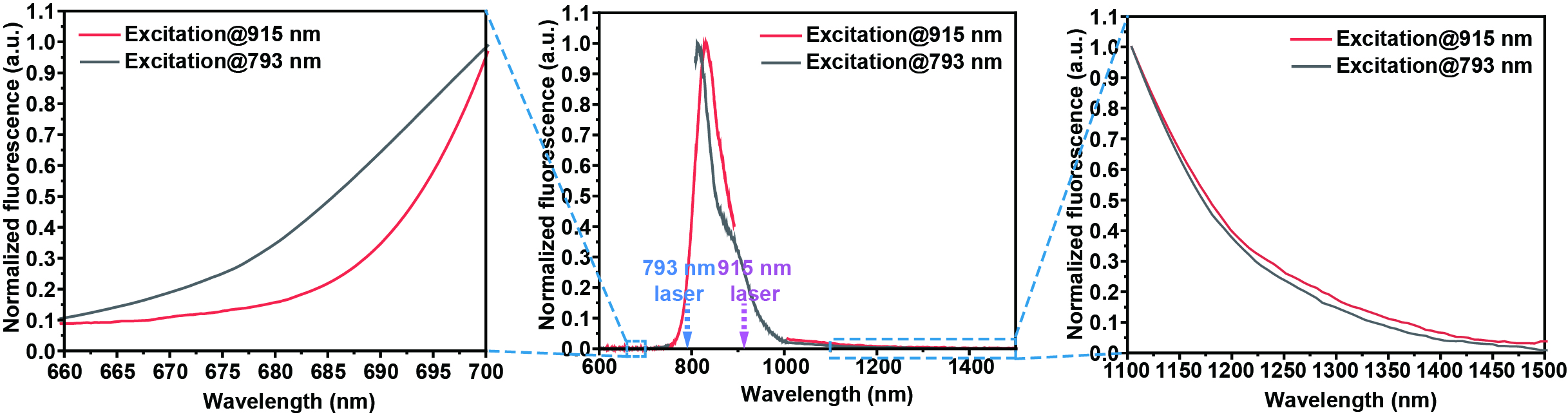


**Fig. S11.** Full fluorescence spectra (600 nm – 1500 nm) of ICG excited by 915 nm and 793 nm CW laser.


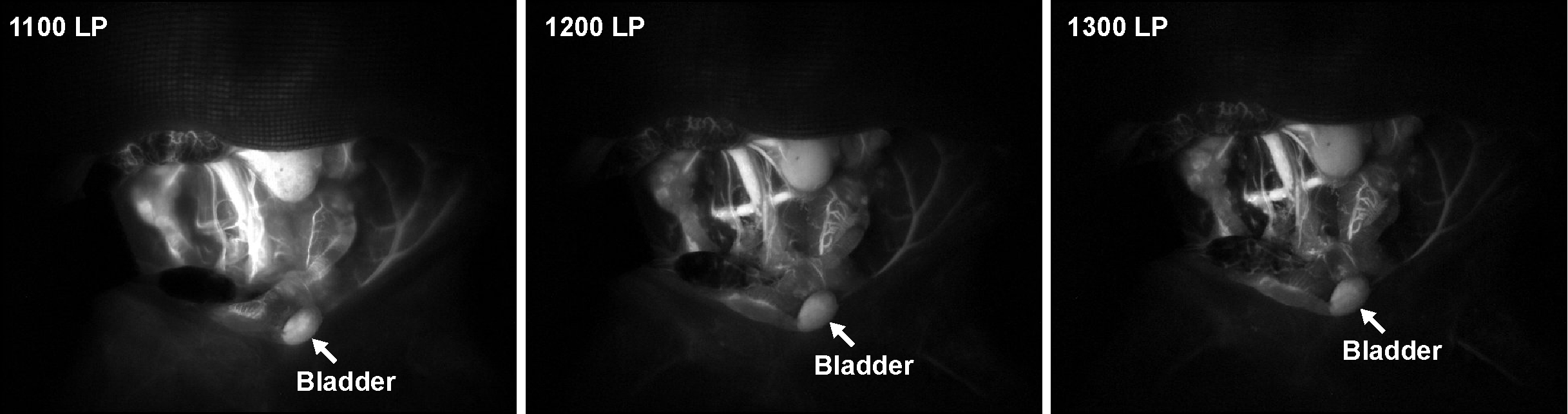


**Fig. S12.** Fluorescence crosstalk of ICG and L1057 NPs in channel 2 under the excitation of 793 nm laser.


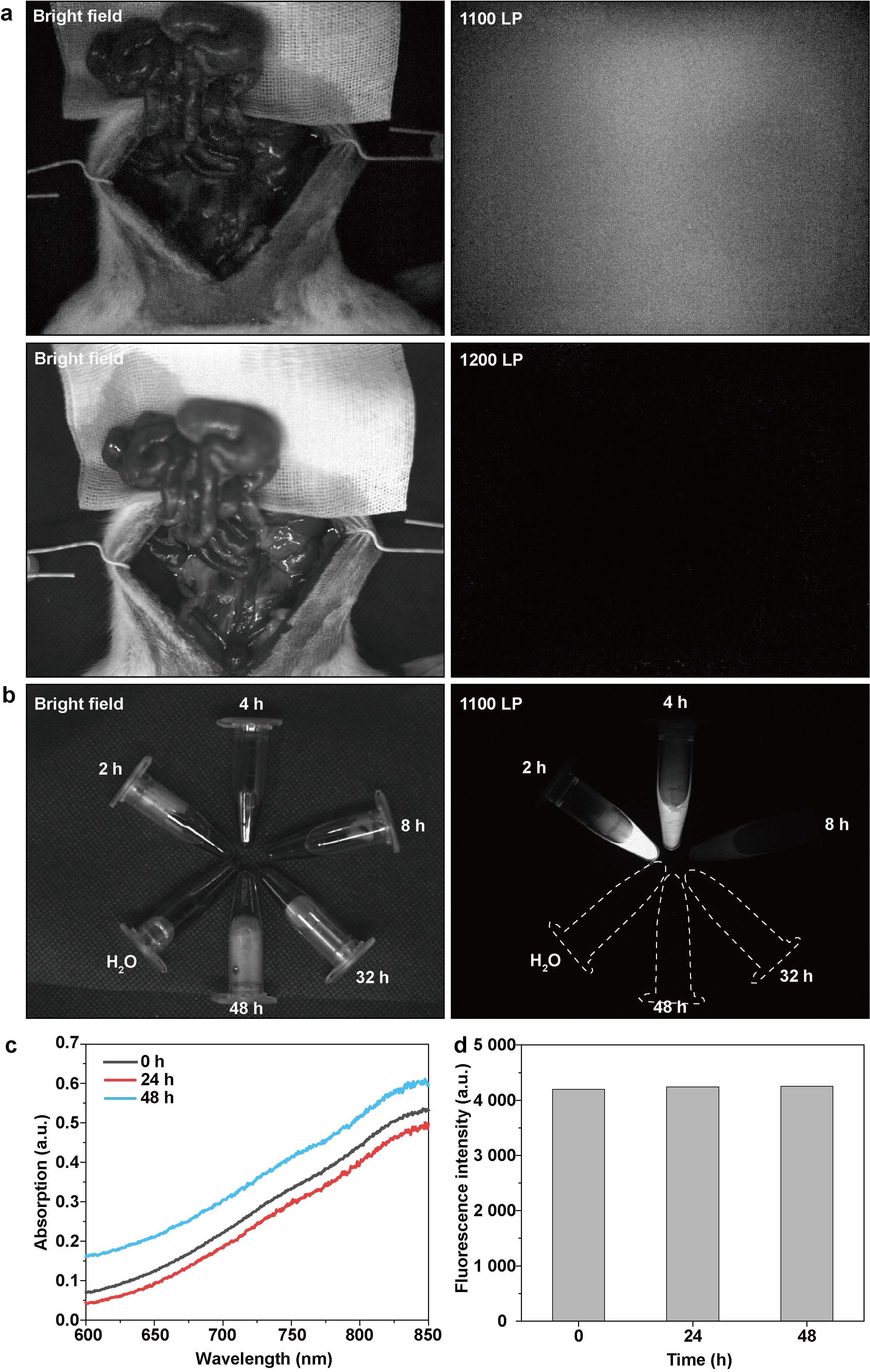


**Fig. S13. Study on the residue of L1057 NPs in the urinary system.** (a) Bright-field and fluorescence images of rat’s urinary system 48 hours after L1057 NPs injection. (b) Bright-field and fluorescence images of rat’s urine collected from 2 hours to 48 hours after the rat was injected with L1057 NPs. Stability analysis of L1057 NPs in rat’s urine taking absorption (c) and fluorescence intensity (d) as references.

**
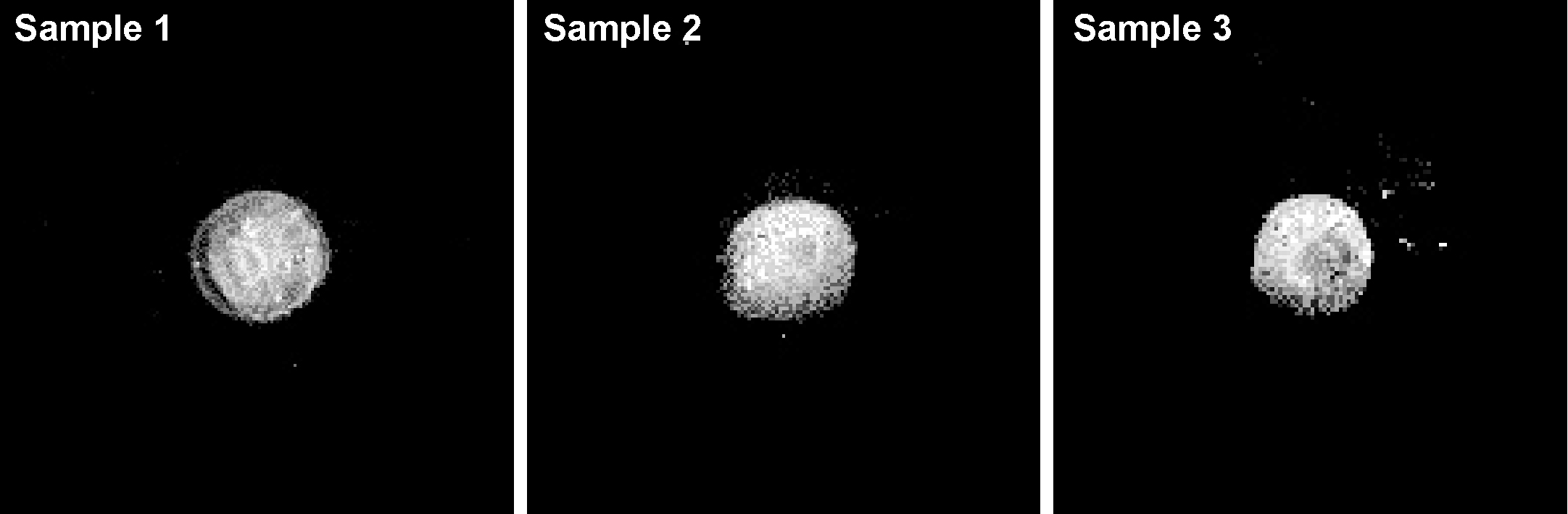
**

**Fig. S14.** ASF (< 700 nm) imaging of three ICG-albumin solid samples under 915 nm CW laser excitation.

**Supplementary video MOV S1 |** Video of monitoring cerebral vascular velocity in a mouse. The imaging was done using a 915 nm CW laser and 800 nm - 900 nm detection. The video here shows dark spots flowing along cerebral vessels. The exposure time was 30 ms. A 25X objective (NA = 1.05) was used.

**Supplementary video MOV S2 |** Video of multi-mode imaging of urinary system and blood vessels under a single 915 nm CW laser excitation. In channel 1 (left), the blood vessels and the urinary system present high contrast visualization, achieving automatic two-organ simultaneous visualization. Synchronously, in channel 2 (right), only urinary system has signals, achieving the single-target visualization.
